# Plasma biomarkers TAP, CPA1, and CPA2 for the detection of pancreatic injury in rat: the development of a novel multiplex IA-LC-MS/MS assay and biomarker performance evaluation

**DOI:** 10.1007/s00204-022-03425-9

**Published:** 2022-12-08

**Authors:** Katerina Vlasakova, Andreas Steinhilber, Wendy J. Bailey, Zoltan Erdos, Hanna Haag, Thomas Joos, Bradley L. Ackermann, Oliver Poetz, Warren E. Glaab

**Affiliations:** 1 Merck & Co., Inc., 770 Sumneytown Pike, WP-45-320, West Point, PA 19486 USA; 2 SIGNATOPE GmbH, Reutlingen, Germany; 3 NMI Natural and Medical Sciences Institute at the University of Tuebingen, Reutlingen, Germany; 4 Eli Lilly and Company,, Indianapolis, IN USA

**Keywords:** Drug-induced pancreas injury, Biomarker, Carboxypeptidase, Immunoaffinity-LC-MS, Trypsinogen activation peptide

## Abstract

Drug-induced pancreatic injury (DIPI) is an issue seen in drug development both in nonclinical and clinical contexts. DIPI is typically monitored by measurement of lipase and/or amylase, however, both enzymes lack sensitivity and specificity. Although candidate protein biomarkers specific to pancreas exist, antibody-based assay development is difficult due to their small size or the rapid cleavage by proteolytic enzymes released during pancreatic injury. Here we report the development of a novel multiplexed immunoaffinity-based liquid chromatography mass spectrometric assay (IA-LC-MS/MS) for trypsinogen activation peptide (TAP) and carboxypeptidases A1 and A2 (CPA1, CPA2). This method is based on the enzymatic digestion of the target proteins, immunoprecipitation of the peptides with specific antibodies and LC-MS/MS analysis. This assay was used to detect TAP, CPA1, and CPA2 in 470 plasma samples collected from 9 in-vivo rat studies with pancreatic injury and 8 specificity studies with injury in other organs to assess their performance in monitoring exocrine pancreas injury. The TAP, CPA1, and CPA2 response was compared to histopathology, lipase, amylase and microRNA217. In summary, TAP, CPA1, and CPA2 proteins measured in rat plasma were sensitive and specific biomarkers for monitoring drug-induced pancreatic injury; outperforming lipase and amylase both by higher sensitivity of detection and by sustained increases in plasma observed over a longer time period. These protein-based assays and potentially others under development, are valuable tools for use in nonclinical drug development and as future translatable biomarkers for assessment in clinical settings to further improve patient safety.

**Supplementary Information:**

The online version contains supplementary material available at 10.1007/s00204-022-03425-9.

## Introduction

Drug induced pancreatic injury (DIPI) can occur in pre-clinical species and in humans during drug development. Acute pancreatitis, an inflammatory disorder of the pancreas, is one of the most frequent gastrointestinal causes of hospital admission (Lankisch et al. 2015; Peery et al. 2012). Even though drug induced pancreatitis accounts for only 0.1-2% of acute pancreatitis cases, the long-term consequences like new-onset prediabetes, diabetes or exocrine pancreatic insufficiency remain substantial risks for the patients (Ismail and Bhayana 2017; Jones et al. 2015; Lee and Papachristou 2019). Diagnosis is usually based on physical signs, imaging techniques and clinical findings of increased serum amylase and lipase (Meher et al. 2015). However, it has been established that although pancreatic enzymatic activity assays for amylase and lipase are important in the diagnosis, they lack sensitivity and specificity in detecting pancreatic injury both clinically and pre-clinically. Additional frequently used biomarkers like C-reactive protein, interleukin-6 and others, reviewed in (Meher et al. 2015), are often general biomarkers of inflammation and therefore lack specificity. In pre-clinical drug development, the lack of sensitive and specific biomarkers remains a concern for monitoring DIPI in pre-clinical species and has been an impediment for advancing drug candidates into the clinic. Recently, it has been demonstrated in in-vivo rat studies that miRNAs, namely miR-217, is sensitive and specific, and persists in circulation longer than amylase and lipase (Erdos et al. 2020).

The physiological function of the exocrine pancreas is to synthesize, store and secrete digestive enzymes and their inactive proenzymes, zymogens. The first step in trypsinogen activation into active trypsin, is the release of trypsinogen activation peptide (TAP). This reaction is normally catalyzed by intestinal enterokinase in the duodenum. Active trypsin then activates zymogens such as chymotrypsinogen, proelastase, prophospholipase and pro-carboxypeptidases converting them into chymotrypsin, elastase, phospholipase and carboxypeptidases, respectively (Frossard 2001; Jones et al. 2015; Kylanpaa-Back et al. 2002). Premature activation of zymogens prior to exiting the pancreatic interstitium results in autodigestion of pancreas tissue and subsequent local inflammation causing acute pancreatitis (Meher et al. 2015; Saluja et al. 2019).

One line of research identifying new biomarkers of exocrine pancreatic injury is focusing on methodologies for detection of pancreatic proteins in blood and urine. The expectation is that these biomarkers will be able to detect pancreatic injury early, with great specificity, and could potentially inform on severity of disease. In the early nineties, urinary TAP was shown to be a promising biomarker for stratifying severity of acute pancreatitis in clinical settings (Gudgeon et al. 1990; Huang et al. 2013; Neoptolemos et al. 2000). Trypsinogen-2, also measured in urine, performed similarly to serum amylase and lipase (Saez et al. 2005). Pancreas-specific carboxypeptidases A1, A2 and B1 (CPA1, CPA2, CPB) are investigated as biomarkers of pancreatic injury, some as pro-carboxypeptidases, active carboxypeptidases or as carboxypeptidase B1 activation peptide (CAPAP). (Kylanpaa-Back et al. 2002; Matsugi et al. 2007; Muller et al. 2002; Pezzilli et al. 2000; Saez et al. 2005). Even though the potential utility of these markers has been shown in a limited number of samples in humans, the limitation for further evaluation both in clinical and in pre-clinical settings is the methodology for their detection due to their small size and/or potential rapid cleavage.

Previously, no assays for measuring TAP were available for rat or human and no CPA1 and CPA2 assays from verified manufacturers were available for rat. Here we report development of a novel multiplexed immunoaffinity-based liquid chromatography mass spectrometric assay IA-LC-MS/MS for detecting TAP, CPA1 and CPA2 in rat plasma. This method is based on the enzymatic digestion of the target proteins, immunoprecipitation of the peptides with peptide-specific antibodies followed by LC-MS/MS analysis. The performance of the biomarkers TAP, CPA1 and CPA2 was evaluated on 470 samples from in-vivo rat studies. The data set included studies with known pancreatic toxicants as well as studies with organ toxicities in liver, kidney, muscle, the gastrointestinal system or vasculature to establish specificity. The performance of TAP, CPA1 and CPA2 was compared to histopathology, lipase, amylase and miR-217 and demonstrated added value for monitoring DIPI in rat plasma.

## Materials and methods

### In vivo rat studies

All studies were approved by the Merck and Co., Inc., Rahway, NJ, USA Institutional Animal Care and Use Committee and conducted in an Association for Assessment and Accreditation of Laboratory Animal Care International–accredited facility in compliance with the National Institutes of Health (NIH) Guide for the Care and Use of Laboratory Animals and the Animal Welfare Act. While most of the animal studies were performed in Sprague-Dawley (SD) rat, some of the studies used Wistar-Han WI(HAN) rats, reflecting company shift to usage of WI(HAN) as a preferable strain for toxicity studies. Both strains we purchased from Charles River Laboratories, Inc. (Raleigh, North Carolina). The animals were acclimated and randomized into treatment and control groups. During the studies, WI(HAN) rats were fed ad lib, and SD animals were maintained on a caloric-optimized diet. The studies were short term, with necropsy time-points ranging from 4 hours to 33 days and the study design often incorporating multiple time-points and doses. All plasma samples were collected as terminal samples at necropsy and had corresponding histopathological examination. Urine was collected only from two studies over night before necropsy on wet ice. The compounds, strain, dose levels, necropsy days, vehicle and route of administration as well as the examined organs and histopathological outcomes are presented in Table 1. Doses were calculated based on animal body weight, and the last dose was given approximately 24 hours prior to necropsy in studies with daily dosing. Blood samples were collected from fasted animals via vena cava and were split into two parts. One part was processed into serum for clinical chemistry analyses and the remaining blood was processed into K3 EDTA plasma for biomarker analyses. Serum was analyzed at the time of necropsy for amylase and lipase; both were measured enzymatically on an automated clinical chemistry analyzer. Plasma was frozen at −70 ºC until subsequent biomarker analyses. Mi-RNA217-5p (miR-217) was assessed on samples previously, using a qPCR method directly from plasma as described (Erdos et al. 2020).

**Table 1:** Study details and histopathology findings

	Strain/dosing/frequency/v ehicle	Tissues examined	Necropsy time (hour or day) /dose in mg/kg	Histopathological findings (grade) [number of animals with finding/total number of animals per group]
Studies with pancreas toxicity				
MRK1-A	SD/oral/daily/A	P,L,K,M,H	6 h/100	Pancreas: atrophy (2) [2/5], edema [4/5], inflammation (1) [3/5], vacuolation (1) [5/5]
			6 h/500	Pancreas: atrophy (1–2) [2/3], edema [3/3], inflammation (1) [3/3], vacuolation (2–3) [3/3]
			D2/100	Pancreas: deg/necrosis (2–3) [5/5], atrophy (2–4) [5/5], edema [5/5], inflammation (3–4) [5/5]
			D2/500	Pancreas: deg/necrosis (2–3) [4/4], atrophy (4–5) [4/4], edema [4/4], inflammation (2–3) [4/4]
			D3/100	Pancreas: deg/necrosis (2–3) [5/5], atrophy (5) [5/5], edema [5/5], inflammation (4–5) [5/5]
			D3/500	Pancreas: deg/necrosis (2–3) [5/5], atrophy (5) [5/5], edema [5/5], inflammation (4–5) [5/5]; liver: necrosis (1) [1/5]
MRK1-B	SD/oral/daily/A	P,L,K,M,H,SI, St,Bl,Co	D3/100	Pancreas: degeneration (3) [4/4], atrophy (1) [4/4], inflammation (3) [4/4]
			D3/500	Pancreas: degeneration (4) [4/4], atrophy (1) [4/4], inflammation (3) [4/4]
			D8/100	Pancreas: degeneration (1–2) [3/3], atrophy (3–4) [3/3], inflammation (2–3) [3/3]
			D8/500	Pancreas: degeneration (1) [1/2], atrophy (2–4) [2/2], inflammation (2–3) [2/2]
Caerulein	SD/IP/4 doses in 1 day/D	P,L,K,M,H	4 h/40	Pancreas: degeneration (1–2) [8/8], atrophy (1) [8/8], inflammation (1) [8/8]
			4 h/120	Pancreas: degeneration (2–3) [8/8], atrophy (1) [8/8], inflammation (1) [8/8]
			D2 40	Pancreas: degeneration (1–2) [8/8], atrophy (1) [7/8], inflammation (1) [4/8], fibrosis (1) [6/8]
			D2 120	Pancreas: degeneration (1–2) [8/8], atrophy (1) [8/8], inflammation (1) [8/8], fibrosis (1) [8/8]
			D4 40	Pancreas: atrophy (1–2) [7/8], inflammation (1) [1/8], fibrosis (1–2) [5/8]
			D4 120	Pancreas: atrophy (1–3) [7/8], inflammation (1) [3/8], fibrosis (1–3) [8/8]
Caerulein + cyclosporin + ETOH	SD/IP, 4 doses in one day */C	P,L,K,M,H	D2/ 0.12	Pancreas: degeneration/necrosis (2–3) [6/6], atrophy (3) [6/6], inflammation (2–3) [6/6]; kidney: degeneration (1) [6/6]; liver: necrosis (1) [1/6]
			D9/0.12	Pancreas: degeneration(1) [6/6],atrophy (1–2) [6/6], fibrosis (1–2) [6/6], islet vacuolization (2) [6/6]; kidney: degeneration (1–2) [4/6]; heart: necrosis (1) [1/6]
			D15/0.12	Pancreas: degeneration (1) [8/8], atrophy (1) [1/8], fibrosis (1–2) [8/8], islet vacuolization (1–2) [8/8]; kidney: degeneration (1–2) [8/8]; heart: necrosis (1) [1/6]
Dibutyltin dichloride	SD/IV/ 1 dose/F	P,L,K,M,H	D2/8	Pancreas: edema-lobular (1–2) [2/9], vacuolation (1) [1/9]; Kidney: tubule necrosis-epithelial (1–2) [3/9]; Liver: focal necrosis (1) [4/9]
			D5/8	Pancreas: atrophy (4) [1/8], edema-lobular (1–2) [4/8], fibrosis (2) [1/8], vacuolation (1) [1/8]; Kidney: tubule necrosis-epithelial (1) [1/8]; Liver: focal necrosis (1–3), [2/8]
			D8-9/8	Pancreas: atrophy (3–4) [3/3],edema-lobular (1–3) [3/3], fibrosis (2–3) [3/3]; Liver: focal necrosis (4) [1/3]
			D14/8	Pancreas: atrophy (3–4) [3/6],edema-lobular (1–2) [2/6], fibrosis (2–4) [3/6]; Liver: focal necrosis (1–4) [3/6]
			D33/8	Pancreas: atrophy (4) [5/8], fibrosis (1–2) [4/8]; Liver: focal necrosis (1) [3/8]
L-arginine	SD/IP/ 1 dose/D	P,L,K,M,H,A	D3/5000	Pancreas: acinus degeneration (1–5) [8/10], inflammation (2) [3/10]
1-cyano-2-hydroxy-3-butene	SD/SC/1dose/D	P,L,K,M,H	6 h/50	Pancreas: peri- and interlobar interstitial edema (2) [1/8]
			6 h/200	Pancreas: peri- and interlobar interstitial edema (3–4) [4/5]
			D2/50	Pancreas: necrosis (1–3) [7/7], atrophy (1–2) [3/7], peri- and interlobar interstitial edema (1) [2/7], vacuolation (2) [1/7]
			D4/50	Pancreas: necrosis (1–3) [7/8], atrophy (1) [1/8]
DL-Ethionine	W/diet/daily/E	P,L,K,M,H,T,	D7/0.25%	Pancreas: degeneration/necrosis (1–3) [8/8], atrophy (1–2)[4/8], inflammation (2) [2/8]; kidney: necrosis (1–3) [8/8]
			D30/0.25%	Pancreas: degeneration/necrosis (2–3) [8/8], atrophy (1)[2/8], inflammation (1–2) [8/8]; kidney: necrosis (3) [1/8]; liver; necrosis(1) [7/8],heart: necrosis(1) [1/8]; testes: degeneration/necrosis (1) [8/8]
Streptozotocin	SD/IV/1dose/B	P,L,K,M,H,T	6 h/30	Pancreas: islets necrosis (1–2) [5/5]
			6 h/60	Pancreas: islets necrosis (3) [5/5]
			D2/30	Pancreas: islets necrosis (1) [2/5], islet decreased size (1) [4/5]
			D2/60	Pancreas: acinus necrosis (1) [1/5], islets necrosis (1) [4/5], islet decreased size (1) [5/5]

Study	Strain/dosing/frequency/vehicle	Tissues examined	Necropsy time (day) /dose in mg/kg	Histopathological findings (grade)
Studies with non-pancreas toxicity				
Acetaminophen (liver)	SD/oral/ 1 dose/A	P,L,K,M,H,SI,LI,St,bone, MA	D2/1000	Liver: necrosis (1–3), inflammation (1)
Bromobenzene (liver)	W/IP/1dose/G	L,K,M,H	D3/300,750	Liver: degeneration/necrosis (1–4), inflammation (1–2)
Carbon Tetrachloride (liver)	W/oral/1daily/G	L,K,M,H	D3,4,8,15/120	Liver: degeneration/necrosis (1–4)
MRK2 (GI)	SD/oral/daily/A	P,L,K,M,H,SI, LI,St	D2,3/100	Duodenum, Epithelium, Single cell necrosis (0–3)
MRK6 (liver, testes)	W/oral/daily/H	P,L,K,M,H, T, SI,brain, thymus	D8/10,50	Bile duct necrosis mucosal (0–4), testis: seminiferous tubule, degeneration (0–2)
Thioacetamide (kidney)	SD/oral/1 dose/I	L,K,M,H	D2/50,100,200	Kidney: tubule degeneration (1–2), liver: degeneration/necrosis (1–4), inflammation (1–3)
Cisplatin (kidney)	SD/IP/1 dose/D	L,K,M,H	D5/10,50	Kidney: tubule degeneration (0–3)
Fenoldopam (vascular)	SD/SC/daily/D	P,L,K,M,H,T,M,A	D2,3,7/5	Mesenteric Vessels, Inflammation, perivascular (0–2), artery, degeneration, medial (0–3); pancreas inflammation perivascular (0–2), artery degeneration, medial (0–3)

Strain: *SD* sprague–dawley, *W* Wistar-Han, Route: *IV* intravenous; *IP* intraperitoneal, *SC* subcutaneous

Vehicle: A 0.5% [w/v] methylcellulose in water; B 4.45 mg/mL trisodium citrate buffer in 0.9% sodium chloride (pH ~ 4.5); C olive oil; D 0.9% NaCl in water; E standard diet; F 40% EtOH 60% glycerol; G corn oil; H 95% Capmul MCM EP/5% polysorbate 80, I water

Tissues: *P* pancreas, *L* liver, *K* kidney, *M* muscle, *H* heart, *T* testes, *SI* small intestines, *St* stomach, *Bl* bladder, *Co* colon, *A* adrenal, *LI* large intestines, *MA* mesenteric artery

^*^Animals received EtOH in the diet from 23 days prior to caerulein injection until necropsy; 20 mg/kg cyclosporin was administered daily from 8 days prior to caerulein injection until the day prior to necropsy

Necropsy was performed and select tissues were processed for histomorphologic examination. Tissue sections were fixed in 10% neutral buffered formalin for approximately 24 hours, processed and embedded in paraffin. Embedded tissues were cut into 4-6 micron sections and stained with hematoxylin and eosin. Stained tissue sections were examined microscopically and severity grades were assigned using a score scale of 0 to 5: 0 (no observable pathology), 1 (minimal or very slight), 2 (mild or slight), 3 (moderate), 4 (marked), or 5 (severe).

### IA-LC-MS/MS assay for quantification of TAP, CPA1 and CPA2

The immunoaffinity-based liquid chromatography tandem mass spectrometry assay (IA-LC-MS/MS assay) comprises enzymatic fragmentation of rat EDTA plasma samples, peptide-targeted immunoprecipitation and peptide separation by nano liquid chromatography with tandem mass spectrometric detection. Calibration was performed with a dilution curve of synthetic reference peptides and ^13^C/^15^N-labeled synthetic peptides as internal standards (INTAVIS GmbH, Tübingen, Germany). Three EDTA plasma samples with known biomarker concentration were used as biological quality control (QC) samples that were carried through the analytical process. Digestion of rat plasma samples was performed in a 96-well microtiter plate. 5 µL of plasma sample, surrogate matrix or QC sample were pipetted into 25 µL digestion buffer (100 mM triethanolamine containing 0.5% n-octylglucopyranoside). Proteins were denatured by heating at 99°C for 5 min and then reduced by adding 5 µL of a 350 mM tris-(2-carboxyethyl)-phosphin (TCEP) solution to reach a concentration of 5 mM TCEP. 5 µL of an 80 mM iodoacetamide (IAA) solution was used for alkylation. Assuming a total plasma protein concentration of 60 mg/mL, 5 µL trypsin-solution with a concentration of 3 mg/mL (15 µg) was added to each sample (approximately 300 µg protein) to give a 1:20 enzyme:plasma protein ratio for enzymatic fragmentation. Digestion was performed overnight at 37°C. Finally, 5 µL of a 200 mM phenylmethylsulfonylfluorid (PMSF) solution was added to stop the reaction (final concentration 1 mM).

Immunoprecipitation of peptides was performed in phosphate buffered saline containing 0.03% 3-((3-cholamidopropyl) dimethylammonio)-1-propanesulfonate (CHAPS) detergent for 1 h. 5 µg of TAP antibody (SIGNATOPE, Reutlingen, Germany) and 2 µg of CPA antibody (SIGNATOPE, Reutlingen, Germany) was added to each well. The antibody-peptide-complexes were precipitated using protein G magnetic microspheres for 1 h. After washing steps in phosphate and bicarbonate buffer, the peptides were eluted from the beads in 1% formic acid.

The LC-MS/MS-analysis of immunoprecipitated peptides was performed on a nano liquid chromatography system (RSLC Ultimate3000, Thermo, Waltham, USA) coupled to a triple quadrupole mass spectrometer (QTRAP 6500+, Framingham, Sciex, MA, USA). The precipitated samples were first trapped on a trap column (0.3 mm I.D. x 5 mm PepMapTM, Thermo, Waltham, USA) and separated on a nano-C18 column (Acclaim Pepmap RSLC C18, 75 μm I.D. × 150 mm, 3 μm, Thermo Fisher Scientific, Waltham, MA, USA). The chromatography was performed as follows: trapping 0.15 min at a flowrate of 150 μL min^-1^, separation 8– 60% eluent B (80% acetonitrile, 20% H_2_O + 0.1% formic acid) in 3.0 min at a flowrate of 1.5 μL min followed by a washing and equilibration step for 2.0 min. All samples were processed in technical duplicates. Peptide detection was performed on a triple quadrupole mass spectrometer equipped with an electrospray ion source (Optiflow, Framingham, Sciex, MA, USA).

The mass spectrometer was operated using multiple reaction monitoring (MRM) in positive mode. Declustering potential was set to 80 V, entrance potential and collision cell exit potential were set to 10 V each. Three transitions per surrogate peptide were monitored and the collision energies were optimized for each transition. The most intense transition was used for data analysis. Unknown concentrations of TAP, CPA1, and CPA2 were calculated from the peak area ratio (analyte/internal standard peptide) using a calibration curve consisting of 8 calibrators (S1-S8) plus blank (B) and a linear fit model. Protein concentrations (ng mL^-1^) were calculated by converting the determined absolute peptide amounts (fmol) considering the molecular weight of the corresponding protein and the analyzed sample volume. The proteins’ molecular weight values were calculated by the amino acid sequence only.

### Quantitative PCR of TAP, CPA1 and CPA2

Primer-probes for the quantification of baseline RNA levels of TAP, CPA1, CPA2, amylase, lipase and two endogenous controls, PUM and GUS in tissue were purchased from Thermo Fisher Scientific (Waltham, MA, USA). TAP (Gene: Prss1; Assay: Rn00754931_m1; cat#4331182), CPA1 (Gene: CPA1; Assay: Rn00566512_m1; cat#4331182), CPA2 (Gene: CPA2, Assay: Rn01500585_m1; cat#4331182), amylase (Gene: Amy2a3; Assay: Rn00821330_g1; cat#4331182), pancreatic lipase (Gene: Pnlip; Assay: Rn00565851_m1; cat#4331182), PUM (Rn00982780_m1; cat# 4331182 ), GUSB (Rn00566655_m1; cat# 4331182). RNA from duodenum, liver and salivary gland was extracted using a Magmax liquid handler and the MagMAX™-96 for Microarrays Total RNA Isolation Kit (all Thermo Fisher Scientific Waltham, MA, USA). Pancreas RNA was extracted with a trizol/chloroform/isopropanol precipitation method to avoid RNA degradation. Reverse transcription of 500 ng total RNA was performed in a 100 µL final volume with the High Capacity cDNA Reverse Transcription Kit (Thermo Fisher Scientific, cat # 4368814) on a Proflex instrument (Thermo Fisher Scientific). Quantitative PCR was performed in triplicate on a Quantstudio 12K Flex in a final volume of 10 µL with 5 µL of 2x Mastermix (Thermo Fisher Scientific cat#4324020), 0.5 µL of 20x Assay on demand (primer-probes) and 2.5 µL water.

### Statistical methods

For the comparison of biomarkers with different plasma concentrations and methods of detection, all measured values were converted to fold changes calculated from the average of each study control group. In cases where measured biomarker values were below the lower limit of quantification (LLOQ), such values were replaced by the lower limit of quantification value. Fold changes from study controls for miR-217 were calculated as (2^(mean control Ct -treated Ct)). Ct = cycle threshold. Samples with no amplification were assigned a Ct value of 40. Mean and standard deviation for each dose-group and time-point were calculated using GraphPad Prism 8.1.1 software.

Receiver operating characteristic (ROC) curves were generated with GraphPad Prism 8.1.1 (Wilson/Brown method); using fold change values calculated from the average of controls of each study. ROC plots provide a statistical method to assess the diagnostic accuracy of a biomarker that has a continuous spectrum of test results. The ROC curve is a graphical display of the trade-offs of the true-positive rate (sensitivity) and false-positive rate (1−specificity) corresponding to all possible binary tests that can be formed from this continuous biomarker. Each classification rule, or cut-off level, generates a point on the graph. The closer the curve follows the left-hand border and then the top-border of the ROC space, the more accurate the test (Soreide 2009). The sensitivity was calculated at 95% specificity where possible. In cases where all controls were below the LLOQ (CPA1, CPA2 and miR-217), and therefore equal to 1-fold change, the sensitivity was calculated at 100% specificity and a positive cut-off value was not determined.

Correlation plots were generated using log10 transformed biomarker fold changes with GraphPad Prism 8.1.1 software, computing Pearson correlation coefficients.

## Results

Since no assays for the quantification of rat TAP, CPA1 and CPA2 were commercially available, we developed IA-LC-MS/MS assays for their detection. Peptide specific polyclonal antibodies towards the TAP peptide APFDDDK and the tryptic CPA1 and CPA2 peptides YSFTFELR and YSFAFELR, respectively, were used to set up IA-LC-MS/MS assays for the human protein variants. Peptide sequences for CPA1 and CPA2 were identical in humans and rats, therefore standards and capture reagents could be used for detection of both. The sequence of TAP differs between rat and human, however, the antibody generated for the human sequence captured the rat variant of the TAP peptide well, and could be used for the analysis for both species. Typical results for calibration curves using the rat peptide sequences are shown in the supplemental material (see Online Resource Figure 1).

### IA-LC-MS/MS TAP and CPA1 and CPA2 assay performance and validation

The assay was validated for performance including the following parameters: limits of quantification, inter and intra assay precision, inter and intra assay accuracy, parallelism, reproducibility, carryover, and stability. A summary of the assay validation results, including parameters with their testing strategy and acceptance criteria, are given in Table 2.

**Table 2:** Assay Validation Parameters with their Testing Strategy, Acceptance Criteria and Results

Parameter	Testing strategy	Acceptance criteria	TAP	CPA1	CPA2
LLOQ	Standard dilution series in surrogate matrix	Accuracy ±25%, precision ≤25%, total error ≤40%	0.537 ng/mL	25.9 ng/mL	25.7 ng/mL
ULOQ			1170 ng/mL	18900 ng/mL	18800 ng/mL
Inter assay accuracy	standard dilution series in surrogate matrix; QC1, QC2, and QC3; three batches	Accuracy ±20% (±25% at LLOQ/ULOQ) for non-zero calibrators and for QC samples (compared to nominal values), total error ≤40%	Passed	Passed	Passed
			Passed	Passed	Passed
			Passed	Passed	Passed
Inter assay precision	Standard dilution series in surrogate matrix; QC1, QC2, and QC3; three batches	Precision ≤20% (≤25% at LLOQ/ULOQ)	Passed	Passed	Passed
			Passed	Passed	Passed
			Passed	Passed	Passed
Intra assay accuracy	QC1, QC2, and QC3; one batch	Accuracy ±20% (±25% at LLOQ/ULOQ) for QC samples (compared to nominal values), total error ≤40%	Passed	Passed	Passed
			Passed	Passed	Passed
Intra assay precision	QC1, QC2, and QC3; one batch	Precision ≤20% (≤25% at LLOQ/ULOQ)	Passed	Passed	Passed
			Passed	Passed	Passed
Parallelism	Eight samples three times serially diluted 1:2 in surrogate matrix	Accuracy ±20% for at least two dilutions in 80% of the samples (e.g. five of six samples)	up to 1:8 dilution	up to 1:8 dilution	up to 1:2 dilution
Reproducibility	Six to ten samples tested in independent two batches	Precision from two or more batches ≤30% in 80% of the samples	Passed	Passed	Passed
Carryover	Standard dilution series in surrogate matrix and blank samples	Blank samples following the calibrators should be below LLOQ	at levels of >391 ng/mL	none	none
Stability	Freeze / thaw stability sample; up to three cycles	Accuracy ±25% of the nominal value	Passed	Passed	Passed
	Short-term stability sample; 2 hours and 24 hours at RT		Passed	Passed	Passed
	Freeze / thaw stability proteolyzed sample; one cycle		Passed	Passed	Passed

The limits of quantification of the IA-LC-MS/MS method for the proteotypic peptides were calculated from three analytical runs. The LLOQ was derived as the lowest concentration level which could be measured with an accuracy ±25%, a mean precision of better than CV ≤25%, and total error of ≤40%. Results for LLOQ were as follows: TAP - 0.537 ng mL^−1^, CPA1 - 25.9 ng mL^−1^ and CPA2 - 25.7 ng mL^−1^, shown in Table 2.

A set of three quality control samples (QC1, QC2, QC3) was used to assess the general assay performance. The quality control samples were prepared by pooling biological samples with high and low endogenous biomarker concentrations to achieve three concentration levels that cover the calibration range at low, medium and high levels. The endogenous concentrations of the proteotypic peptides and the actual protein biomarker concentration in these samples was determined by analyzing those in three independent runs. These values were used as nominal concentrations in the assessment of assay accuracy/precision and analyte stability.

Accuracy and precision data for calibrators and QC samples are reported for TAP, CPA1 and CPA2 in the supplemental material (Online Resource Tables 1–12). The three analytes passed the accuracy and precision criteria for calibrators (inter batch accuracy and precision) and QCs (inter and intra accuracy and precision). Typical results for standard curves are shown in Online Resource Figure 1. TAP passed all concentration levels covering standards S1-S8. CPA1 and CPA2 passed the concentration range for standards S1-S7. Representative extracted ion chromatograms for TAP, CPA1, and CPA2 at the LLOQ, in a blank and the three QC samples are shown in Online Resource Figures 2, 3, and 4.

QC samples subjected to the stability test conditions were analyzed in duplicate per stress time interval. The stability test samples were compared with the nominal values of the QCs. If the accuracy was ±25%, the samples were considered stable. The results of the stability validation are shown in supplemental material (Online Resource Tables 13–15). TAP, CPA1, and CPA2 passed the freeze-thaw-stability of non-processed samples (−80 °C storage) for all QCs. All three analytes were stable in non-processed samples stored at room temperature for 2 hours and 24 hours before digestion. The analytes were stable after proteolysis in one freeze-thaw-cycle (−20 °C storage). Moreover, immunoprecipitated TAP, CPA1, and CPA2 peptides were stable for up to 72 hours in the autosampler, since results of re-injections after 24 hours, 48 hours and 72 hours (5 °C storage) matched their nominal QC values (Online Resource Tables 13-15).

### Biomarker analyses

TAP, CPA1, and CPA2 were analyzed in a total of 300 plasma samples from 9 in-vivo rat studies treated with pancreatic toxicants. Specificity was confirmed with samples from the streptozotocin study (damaging only beta cells in pancreatic islets; 30 samples) and 8 studies (140 samples) with toxicities in liver, kidney, GI or vasculature. Urine was available only from MRK-B study with pancreas toxicity and one liver specificity study. Study details including route of administration, dosing frequency, vehicle, tissues histopathologically examined, and detailed histopathological findings for each dose group and time-point are summarized in Table 1.

**Fig.1:**
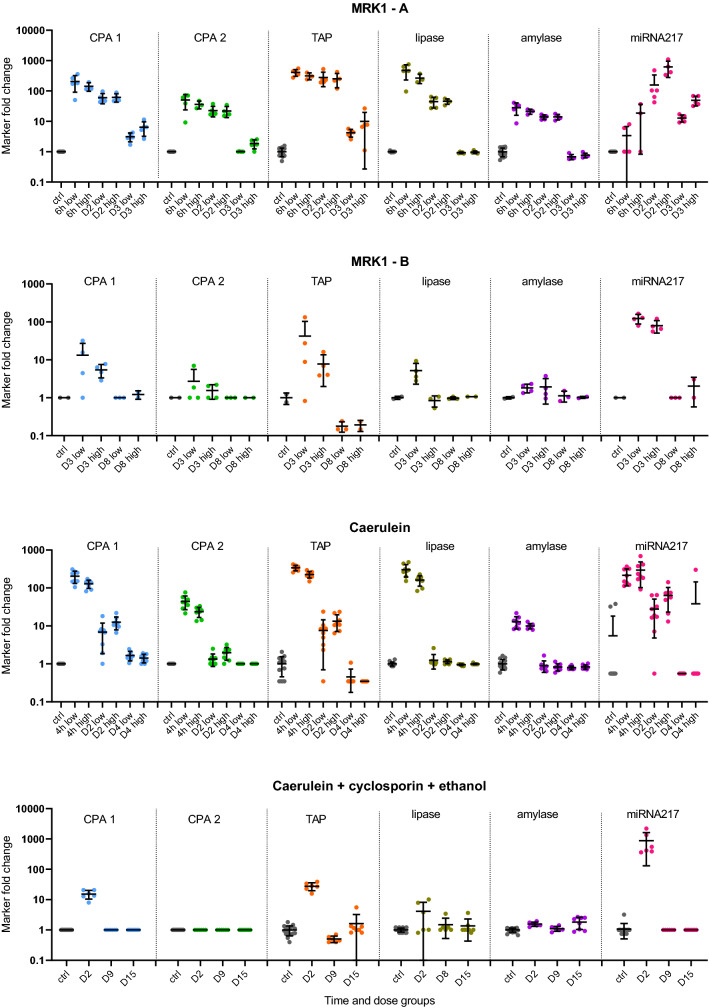

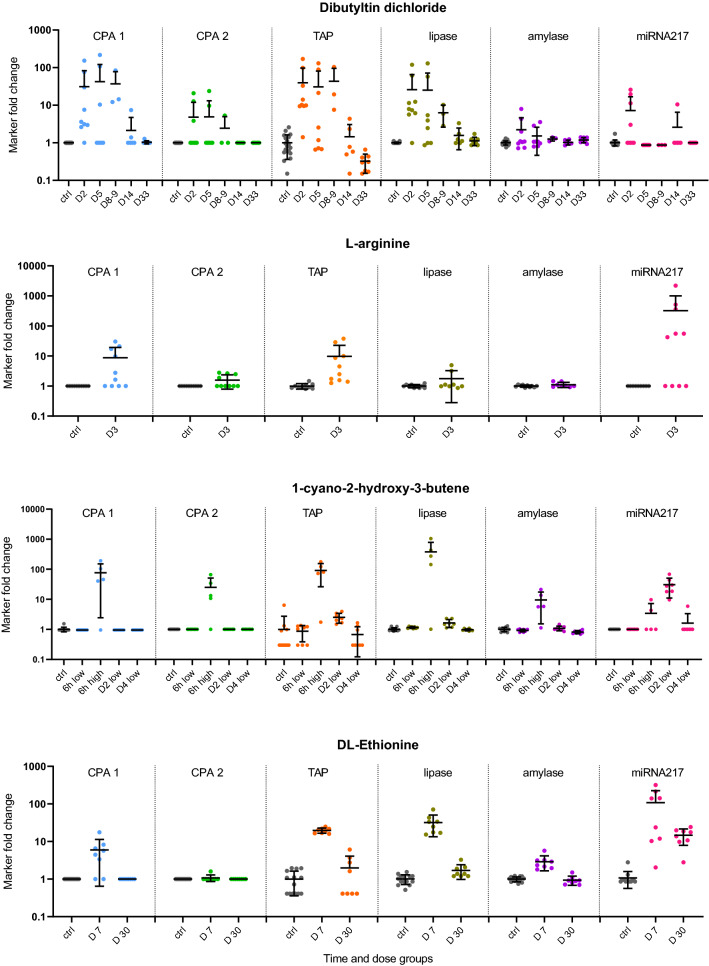
Biomarker performance across in-vivo studies. Biomarker values are shown as fold changes from each study control group. Mean and standard deviation for each time and dose-group are shown. Time and dose-groups follow the same order as in Table 1, where detailed histopathological findings for each group are listed. LLOQ values were replaced by the LLOQ and resulting fold changes for such samples was calculated as 1-fold

A list of biomarker candidates was first selected from differential bottom-up global proteomics experiments (data not shown), supported by literature, and evaluated in a smaller sample set with acinar cell specific toxicants. The final three biomarkers were selected based on their performance and response to acinar cell toxicity and then assessed in a larger study set, evaluating both sensitivity and specificity. TAP, CPA1, and CPA2 performance in studies with pancreatic toxicants is summarized in Fig. 1 and Table 3. In Fig. 1, each biomarker value is expressed as fold change from the average of the controls from each respective study. Samples are organized to follow the same time- and dose-groups order as in Table 1, where detailed histopathological findings for those groups are listed. Mean and standard deviation for each dose-group and time-point are also shown. Table 3 provides a summary overview of the alignment of the studies histopathological findings expressed as percent of animals in a time- and dose-group where specific histopathological findings are listed with the average fold change of the given biomarker in that group. For better visualization, the histopathological findings and biomarker fold changes are color coded. In the specificity studies including the streptozotocin study, none of the three biomarkers changed consistently, in fact, values for CPA1 and CPA2 were below the LLOQ in all specificity samples and therefore data from the specificity studies are not presented in this table.

**Table 3:**
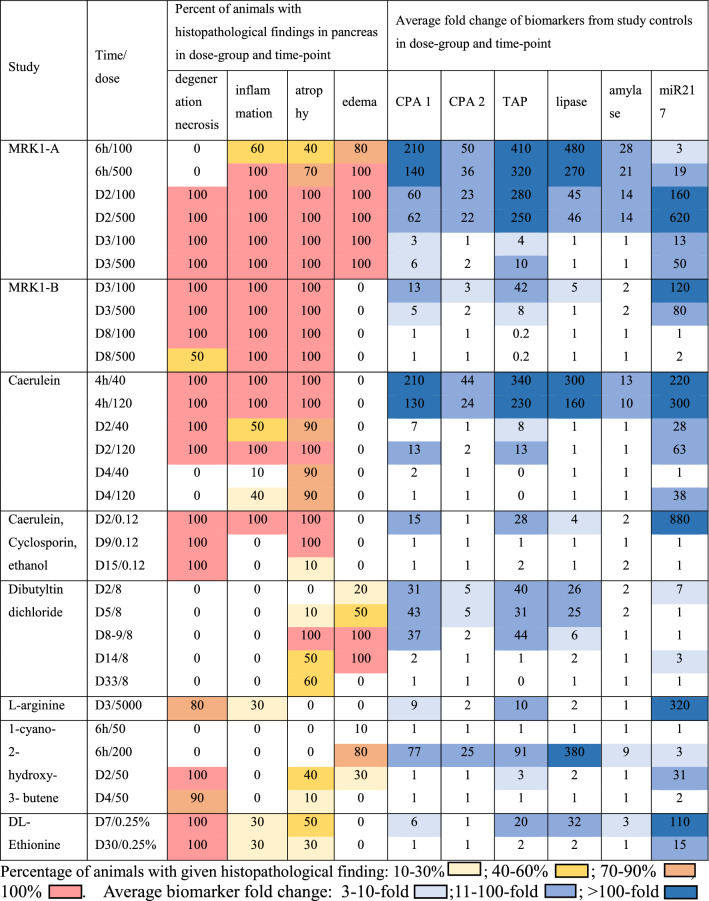
Summary overview of histopathological findings in pancreas and average biomarker fold change

MiR-217 was used, together with activity assays amylase and lipase, as a comparator for TAP, CPA1, and CPA2 based on its performance in rat studies with pancreatic injury (Erdos et al. 2020), where several miRNAs were evaluated and miR-217 was selected as the best performer for its sensitivity and specificity for pancreatic injury.

### ROC curve and correlation analyses

**Fig 2:**
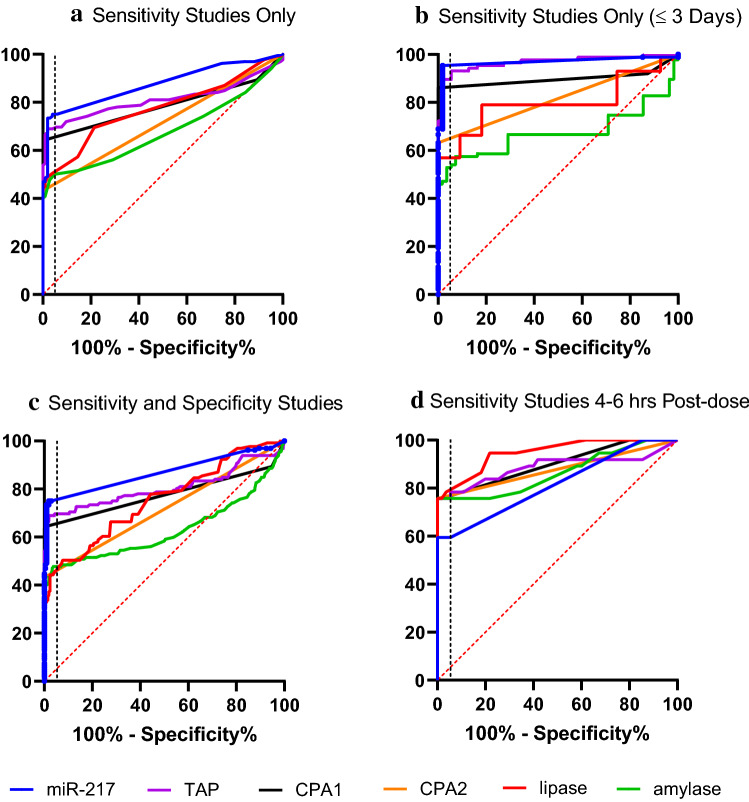
Receiver operating characteristics (ROC) curve analyses. Exclusion models presented. Description and results for each analysis is summarized in Table 4.

ROC curve analyses were conducted to allow for performance comparison between individual biomarkers. The area under the curve (AUC) provides an assessment of overall performance. Results of ROC curves and analyses with the AUC and % sensitivity at 95%-100% specificity are summarized in Table 4 and visualized in Fig.2A–D. To gain more detailed insight into the biomarker performance relative to the time from the initial dose, four separate analyses were performed. For these analyses, an exclusion model was utilized, where animals treated with pancreas toxicants that did not present with histopathologic findings were excluded from the analyses. The first analysis conducted was on samples with histopathologically confirmed pancreatic injury (defined as acinar cell degeneration/necrosis/inflammation) and contained 103 controls and 132 treated samples (Fig. 2A). In this analysis, study samples treated with toxicant, but without pancreatic injury were excluded. MiR-217 had the highest AUC (0.87) and sensitivity (75%), due to the longer presence in plasma compared to the other biomarkers. In the group of the pancreatic enzymes, TAP was the best performer (AUC 0.81, 69% specificity) followed by CPA1 (AUC 0.79, 65% specificity). A second analysis focused on the same sample set but was limited to samples collected up to 72 hours post-dose (total of 55 controls and 87 treated samples) to gain insight into the biomarker performance during the early stages of acute toxicity (Fig. 2B). For all the biomarkers, the performance characteristics improved. TAP AUC increased from 0.81 to 0.97 and sensitivity from 69 to 90% indicating that the biomarkers perform the best in the acute phase of the injury. In both analyses CPA1 and TAP outperformed amylase and lipase in both AUC and sensitivity. To address the specificity of the biomarkers, an additional ROC analysis was performed using sensitivity studies from 2A with the addition of all specificity study samples (with toxicity in organs other than pancreas and the streptozocin study with beta cell toxicity) to the analysis (Fig. 2C). The performance characteristics did not change, indicating that the biomarkers are increased only with pancreatic injury and not affected by toxicities in other tissues. In fact, all the CPA1 and CPA2 values were below LLOQ in all samples from specificity studies. The fourth analysis shown in Fig. 2D compared results from samples collected from the most acute phase, 4–6 hours post-dose. All samples available for this time-group were included, irrespective of the histopathological findings to account for processes that may release pancreatic enzymes into the bloodstream before histopathological changes are microscopically evident. The sample set was limited to 37 samples from treated animals. Since the control group did not have enough representative samples, it was supplemented by controls from samples up to 72 hours post-dose. In this analysis, TAP, CPA1, CPA2, amylase and lipase outperformed miR-217, likely due to their earlier release into the blood. All the four pancreatic enzymes and the activating peptide were positive in the same samples, as demonstrated by the equal sensitivity.

**Table 4:** Receiver operating characteristics (ROC) curve analyses

		CPA1	CPA2	TAP	amylase	lipase	miR217
Correlated to pancreatic injury							
2. A Sensitivity studies only. All time-points (103 control, 132 treated)	AUC	0.79	0.72	0.81	0.67	0.77	0.87
	sensitivity	64%	43%	69%	50%	50%	75%
	cut-off	N/A	N/A	2	1.3	1.3	N/A
2. B Sensitivity studies only ≤ 3 days (55 controls, 87 treated)	AUC	0.9	0.82	0.97	0.68	0.80	0.97
	sensitivity	86%	63%	90%	53%	57%	95%
	cut-off	N/A	N/A	2.2	1.4	0.3	N/A
2. C Sensitivity and specificity studies. All time-points. 2.A sample set with added specificity samples (40 controls and 68 treated)	AUC	0.78	0.72	0.80	0.63	0.75	0.87
	sensitivity	64 %	43%	70%	48%	46%	75%
	cut-off	N/A	N/A	2	1.4	1.5	N/A
Correlated to treatment with pancreatic toxicant, 4-6 hrs post-dose							
2. D Sensitivity studies 4-6 hours post-dose with controls ≤ 3 days (57 controls, 37 treated)	AUC	0.90	0.88	0.89	0.86	0.95	0.81
	sensitivity	76%	76%	76%	76%	76%	59%
	cut-off	N/A	N/A	1.9	1.4	1.3	N/A

**Fig. 3:**
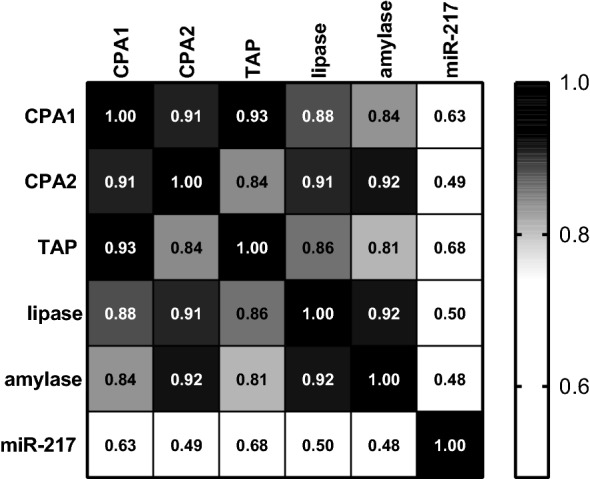
Biomarker Correlation. Values represent correlation coefficients based on biomarker responses from sensitivity study samples. The color scale ranges from black (1.0, high correlation) to white (0.5, low correlation.

Additionally, a correlation analysis was performed to quantify how the performance of the individual biomarker responses are correlated to the other biomarker candidates. The results are presented in Fig. 3. Not surprisingly, the correlation between all the pancreatic enzymes is strong (*r* = 0.84–0.93). Correlation of the miR-217 with the pancreatic enzymes is weaker, confirming different timing of the biomarker presence in the blood. TAP and CPA1 have the best correlation (*r* = 0.93). Amylase and lipase also correlate well (0.92).

### TAP performance

TAP was the best-performing protein biomarker in the ROC analyses comparing performance against acinar cell degeneration necrosis and inflammation up to 3 days (Table 4 and Fig. 2). The sensitivity at 95% specificity reached 90% in the up to 3 days analysis, outperforming lipase, amylase as well as CPA1 and CPA2, but not outperforming miR-217. Unlike CPA1 and CPA2, TAP measurements were quantifiable by the IA-LC-MS/MS method in the majority (80%) of the plasma samples. Fold changes reached 100-fold in magnitude and were the largest at the earliest collection time-points (4–6 hours) in the MRK1-A and caerulein studies. From this early peak, the TAP levels generally decreased over time but were still elevated at SD2 and/or SD3. In the dibutyltin dichloride study, TAP was increased in samples without histopathological findings or with findings of pancreatic edema and/or atrophy at SD2-9, decreased at SD14 and returned to control levels at SD33. In the DL-ethionine study, TAP was increased at SD7 where all animals presented with pancreas degeneration and necrosis but returned close to control levels at SD30.

In the specificity studies, TAP was increased above 3-fold only in 2 samples, both in the MRK6 study. Interestingly, lipase was increased in those samples as well. Those two samples presented with common bile duct mucosal necrosis, grade 2 and 3. Pancreas was histopathologically examined and was found to be unremarkable.

When compared to lipase and amylase, TAP had a similar time-course but outperformed lipase and especially amylase in several dose-groups. In the MRK1-A and MRK1-B studies, TAP was still detectable at SD3 while lipase and amylase were back down to the control levels. In the caerulein study, lipase and TAP were increased similarly (> 100-fold) at 4 hours in both dose groups. However, lipase was undetectable at SD2, while in most of the samples, TAP was still increased (Fig. 1). Performance comparison with miR-217 did show that miR-217 was often not consistently increased at the early time-points (<24 hours), as in the MRK1-A study. However, miR-217 was present as the only marker at SD30 in the DL-ethionine study with corresponding findings of degeneration, necrosis and inflammation. On the other hand, TAP was more sensitive than miR-217 in the dibutyltin dichloride study on SD5 and SD8-9, concomitant with findings of pancreas atrophy, edema and fibrosis (histopathology changes other than acinar cell necrosis/degeneration) as shown in Fig. 1 and Table 3.

### CPA1 and CPA2 performance

CPA1 was the second-best performer, out-performing lipase and amylase in correlation to pancreatic injury. Plasma levels were undetectable in all vehicle-treated control samples as well as in all specificity samples regardless of treatment status. However, CPA1 concentration increased above LLOQ after treatment with compounds causing DIPI. The highest increases reached >100-fold and were observed after 4 hours in the caerulein study (Fig. 1). It is difficult, however, to correctly assess the magnitude of change due to the inability to quantify the CPA1 levels in controls. CPA1 time and dose-related response to injury is similar to the TAP response in most of the sensitivity studies, however, TAP outperformed CPA1 in the DL-ethionine study. CPA2 was the least sensitive performer from the three measured biomarkers. In the ROC comparisons across all study days, the sensitivity was lower than that of lipase and amylase. As observed with CPA1, CPA2 was below the LLOQ in all control and specificity samples. In some dose-groups where CPA1 and TAP were detectable, CPA2 fell below LLOQ as demonstrated at SD2 of the caerulein-cyclosporin-ethanol study and in SD7 of the DL-ethionine study.

### TAP, CPA1 and CPA2 performance in urine

Our analyses were focused on biomarker performance in plasma, however we also evaluated TAP, CPA1, and CPA2 in urine from the MRK1-B study, where plasma TAP and CPA1 were increased in almost all samples at SD3, and in the bromobenzene specificity study presenting with liver toxicity. Urinary TAP was undetectable in most of the tested samples and was increased only slightly in 3 samples in the MRK1-B SD3 group. Urinary CPA1 was detectable in approximately half of the tested samples, and was increased only in one sample of the MRK1-B study with the highest CPA1 plasma concentration. Urinary CPA2 was below LLOQ in a majority of the samples and samples with detectable levels did not correlate with the treatment groups or CPA2 plasma levels (data not shown).

### Tissue specificity and relative abundance

The tissue specificity and relative abundance of TAP, CPA1, and CPA2 was investigated using both protein and mRNA endpoints. Gene expression was evaluated using RNA isolated from recently frozen control tissues (mass-balanced pools consisting of *n*=3 males and *n*=3 females). Quantitative PCR demonstrated the presence of these 3 transcripts predominately in pancreas, while the tissue with the next closest abundance, was duodenum at approximately a 500-fold lower mRNA concentration (Table 5). Other tissues did not have considerable expression, supporting the pancreas specific expression of these biomarkers. Relative abundance was then determined by comparing mRNA copy number in pancreas. The relative abundance was as follows: amylase > TAP > CPA2 ~ lipase > CPA1, with a maximal difference of 10-fold between amylase and CPA1 (Table 5). Specificity was further investigated using protein extracts from a comparable tissue panel and assessed by the multiplex IA-LC-MS/MS assay developed here. Similar findings were observed, confirming pancreas tissue specificity of these biomarkers, however, the relative abundance was slightly different to that of the qPCR assessment: CPA1 > TAP > CPA2, with a maximal difference of 5-fold between CPA1 and CPA2 (data not shown). Regardless of these slight differences in relative abundance, the pancreas specificity for these three biomarkers was clearly demonstrated.

**Table 5.** Baseline RNA copy number in selected control tissues

Tissue	CPA1	CPA2	TAP	Amylase	Lipase
Pancreas	2.0E+07^a^	4.5E+07	8.0E+07	1.9E+08	4.2E+07
Duodenum	4.2E+04	9.9E+04	2.1E+05	3.5E+05	8.4E+04
Liver	2.7E+04	8.8E+03	5.4E+00	3.1E+02	4.5E+00
Salivary gland	7.1E+00	3.2E+01	1.1E+01	4.7E+00	7.2E+00

^a^Copy number per 10 ng of cDNA input

## Discussion

A novel assay for detection of TAP, CPA1 and CPA2 in plasma was developed. Unlike traditional assays that use antibodies targeting parts or the whole protein, this assay uses antibodies against small peptides generated after sample digest with trypsin to enrich for the target by immunoprecipitation. The specificity for the given target is achieved by the specificity of the antibody, the chromatography step and the following mass spectrometric detection. This approach is useful in the case of short peptides like TAP and possibly for other pancreatic enzymes that can undergo cleavage by activated trypsin during the development of DIPI. Measurement of only short peptides may be the reason for longer detection in blood compared to amylase and lipase, both enzymatic assays, that require functional protein to be able to cleave the substrate for measurement. Kinetics of the candidate biomarkers, however, have not been established at this time.

Published studies evaluated TAP as a biomarker mostly in urine using competitive immunoassays (Neoptolemos et al. 2000) or radioimmunoassay (Neoptolemos et al. 2000; Petersson and Borgstrom 2006; Schmidt et al. 1992). While both activity and antibody-based assays are available for human CPA1, validated assays are not available for rat. Similarly, rat assays for CPA2 measurements from verified vendors are also not available. Therefore, the assays developed here offer the possibility to measure and evaluate the performance of the potential biomarkers of DIPI in rat in a multiplexed format. Moreover, the method also has the potential to expand the biomarker selection further to include additional candidates like carboxypeptidase B1 activation peptide (CAPAP) when immunoprecipitation antibodies are generated.

The newly developed assays were evaluated for performance in a set of studies with various pancreas toxicants as well as for specificity in studies with other organ tissue injuries. The in-vivo study sample set was slightly down-selected from the set previously published to evaluate performance of pancreas-specific miRNAs (Erdos et al. 2020). The stability of the biomarkers is unknown, however, the majority of the samples analyzed and presented here were collected from studies performed over 10 years ago, with samples in storage (−70 ^°^C) for that duration, proving that treatment-relevant increases in plasma for TAP, CPA1, and CPA2 can be seen even after prolonged storage. TAP was the best performer of the three protein biomarkers. The majority of published literature focused on measuring TAP in urine due to the observed fast clearance from plasma into urine (Frossard 2001; Huang et al. 2013; Neoptolemos et al. 2000). It seems from our findings, however, that TAP is sufficiently stable in blood to warrant future investigation into the usefulness of this peptide measurement in serum or plasma. The lack of response in urine measured by the IA-LC-MS/MS assay will need to be further evaluated because urine was available from only one study with pancreatic injury after extended storage. It is not clear how much stability in urine has impacted the biomarker performance results reported here. From previous studies analyzing urinary samples collected from pancreatitis patients (Petersson and Borgstrom 2006) it was reported that the full TAP octapeptide (APFDDDDK) is present only in small amounts, while the majority occurs as pentapeptide DDDDK. The rat TAP peptide sequence is FPLEDDDK, similar to the human peptide (Chen et al. 2003) therefore the same immunoprecipitation antibody can recognize both the human and rat peptides. Only modification of the calibrator to be species-specific would be needed. However, it may be the case that the rat peptide is also further degraded to shorter peptides, as described for humans, and that those were missed during quantification, since we did not monitor these shorter fragments. It would be possible to monitor potential smaller fragments in the future when suitable samples become available.

Since TAP is released during the first step of trypsinogen activation, it has the potential to be a very early indicator of DIPI. Indeed, the largest increases were seen hours after the dosing in our studies as well as in other models of rat pancreatitis (Schmidt et al. 1992; Wang et al. 2001), where TAP was measured both in plasma and urine and proved to be a useful biomarker of acute pancreatic injury in rat.

Rat CPA1 and CPA2 are closely related products of genes that duplicated before the mammalian radiation. They hydrolyze peptide bonds of C-terminal residues with aromatic or aliphatic side-chains. CPA1 substrate preference is toward smaller amino acids while CPA2 is towards bulkier amino acids (Gardell et al. 1988). Both are secreted from the pancreas in inactive procarboxypeptide forms. Through the action of trypsin, enzymatically active carboxypeptidase and activation peptide are released (Jimenez et al. 2003). Due to the lack of assays to measure CPA1 in rat, the only information about its performance is from human data and even that is very limited. It was reported that the active form of CPA1, measured as an activity assay, does correlate with pancreatitis, while total CPA (pro carboxypeptidase A plus active CPA) could be useful for detection of both pancreatitis and pancreatic carcinoma. (Matsugi et al. 2007; Shamamian et al. 2006).

The advantage of all three evaluated biomarkers is their predominant presence in pancreas, assuring specific responses to pancreatic injury. Our company created an mRNA body atlas from more than 50 rat tissues and cell lines which confirm the abundance of the TAP, CPA, and CPA2 RNA in pancreas with little to no expression in other tissues (data not shown). Similarly, publicly available rat gene expression data from many tissues and cell lines show predominant expression in the pancreas (Wu et al. 2009). We have observed small amounts of the biomarkers in duodenum, however it is possible that this is due to tissue contamination during tissue collection. The Human Protein Atlas that includes duodenum as an examined tissue, does not list any of the biomarkers as expressed in duodenum (Thul and Lindskog 2018; Uhlen et al. 2015).

Evaluation of the biomarkers in pre-clinical settings provides many advantages such as usage of different dose levels, timing of blood collections, and most importantly, a direct link to histopathological outcomes. One of the disadvantages is that animal models may not accurately recapitulate the events in patients (Gorelick and Lerch 2017). The Translational Safety Biomarker Pipeline (TransBioLine) Project of the IMI2 consortium aims to evaluate and qualify novel safety biomarkers to monitor drug-induced pancreatic injury, including TAP, CPA1, and CPA2 for use in clinical trials. The work presented here provides supporting data sets to further enable the clinical qualification of pancreatic biomarkers.

In summary, the development of a new assay for detection of rat pancreatic proteins allowed the evaluation of their performance as potential biomarkers of DIPI. The drug induced responses of TAP, CPA1, and CPA2 were compared to histopathology, lipase, amylase and miR-217. All three protein biomarkers were increased as early as 4-6 hours post dose, and correlated well with pancreatic injury up to study day three (AUC 0.79-0.97), but were not consistently increased in later stages of pancreatic injury progression. TAP was the best performer of the three markers, out-performing lipase and amylase, but not miR-217 except at early time-points, often prior to acinar cell degeneration/necrosis. None of the protein biomarkers were increased in any of the specificity studies or detected in other organ homogenates, confirming their pancreas specificity. Biomarker analyses using this new assay can be multiplexed and expanded for evaluation of additional rat biomarkers candidates. The IA-LC-MS/MS assay can be easily modified to target peptides in other species providing translational biomarkers from pre-clinical space to the clinic. This would provide additional biomarkers to monitor DIPI in combination with amylase, lipase and miRNA biomarkers, with potential clinical use, further assuring patient safety.

## Supplementary Information

Below is the link to the electronic supplementary material.

Supplementary file1 (DOCX 624 KB)

## Data Availability

The datasets from the analyses of in-vivo studies are available from the corresponding author on reasonable request.
