# Supplementary material for: Plasma biomarkers TAP, CPA1, and CPA2 for the detection of pancreatic injury in rat: the development of a novel multiplex IA-LC-MS/MS assay and biomarker performance evaluation
Source: Arch Toxicol. 2022 Dec 8;97(3):769–85. doi: 10.1007/s00204-022-03425-9 (PMC9968696; doi:10.1007/s00204-022-03425-9)
Supplement: Supplementary file 1 — Supplementary file1 (DOCX 624 KB) [file 204_2022_3425_MOESM1_ESM.docx]

Supplemental Material

Plasma Biomarkers TAP, CPA1, and CPA2 for the Detection of Pancreatic Injury in Rat: The development of a Novel Multiplex IA-LC-MS/MS Assay and Biomarker Performance Evaluation

Katerina Vlasakova*^1,2^, Andreas Steinhilber*^1,3^, Wendy J. Bailey^2^, Zoltan Erdos^2^, Hanna Haag^3^, Thomas Joos^3,4^, Bradley L. Ackermann^5^, Oliver Poetz^3,4^, Warren E. Glaab^2^

^1^Contributed equally to the manuscript

^2^Merck & Co., Inc., West Point, Pennsylvania, USA

^3^SIGNATOPE GmbH, Reutlingen, Germany

^4^NMI Natural and Medical Sciences Institute at the University of Tuebingen, Reutlingen, Germany ^5^Eli Lilly and Company, Indianapolis, Indiana, USA

**Fig.1** Calibration curves of protein biomarker surrogate peptides in matrix. Results are plotted as mean of three batches in duplicates (n=6).

| Blank | LLOQ | QC1 | QC2 | QC3 |
| --- | --- | --- | --- | --- |
|  |  |  |  |  |

**Fig. 2** Representative LC-MS/MS signals of blank, LLOQ, QC1, QC2, QC3 for TAP. Quantitative analysis was performed using the y7++ ion

| Blank | LLOQ | QC1 | QC2 | QC3 |
| --- | --- | --- | --- | --- |
|  |  |  |  |  |

**Fig. 3** Representative LC-MS/MS signals of blank, LLOQ, QC1, QC2, QC3 for CPA1. Quantitative analysis was performed using the y6+ ion.

| Blank | LLOQ | QC1 | QC2 | QC3 |
| --- | --- | --- | --- | --- |
|  |  |  |  |  |

**Fig. 4** Representative LC-MS/MS signals of blank, LLOQ, QC1, QC2, QC3 for CPA2. Quantitative analysis was performed using the y6+ ion.

### TAP accuracy and precision

**Table 1** Overview of accuracy and precision experiments for TAP (n=6)

| Sample set | Parameter | Acceptance criteria | Range within criteria | Min (%) | Max (%) |
| --- | --- | --- | --- | --- | --- |
| S1-S8 | Inter batch precision (%CV) | ≤20%, ≤ 25% at LLOQ | S1-S8 | 4 | 19 |
| S1-S8 | Inter batch accuracy (%accuracy) | ≤±20% | S1-S8 | -8 | 7 |
| S1-S8 | Inter batch total error (%TE) | ≤40% | S1-S8 | 7 | 24 |
| QC1-3 | Intra batch precision (%CV) | ≤20%, ≤ 25% at LLOQ | QC1-3 | 2 | 4 |
| QC1-3 | Intra batch accuracy (%accuracy) | ≤±20% | QC1-3 | -6 | 3 |
| QC1-3 | Intra batch total error (%TE) | ≤40% | QC1-3 | 4 | 10 |
| QC1-3 | Inter batch precision (%CV) | ≤20%, ≤ 25% at LLOQ | QC1-3 | 5 | 7 |
| QC1-3 | Inter batch accuracy (%accuracy) | ≤±20% | QC1-3 | -1 | 3 |
| QC1-3 | Inter batch total error (%TE) | ≤40% | QC1-3 | 7 | 9 |

**Table 2** Inter batch accuracy and precision, calibration samples TAP (n=6)

| Run | Unit | S8 | S7 | S6 | S5 | S4 | S3 | S2 | S1 |
| --- | --- | --- | --- | --- | --- | --- | --- | --- | --- |
| 610090 | ng/mL | 0.52 | 1.71 | 4.97 | 15.01 | 42.93 | 135.86 | 360.29 | 1051.39 |
|  | ng/mL | 0.54 | 1.65 | 4.76 | 14.74 | 44.06 | 130.62 | 386.96 | 1085.22 |
| 610091 | ng/mL | 0.65 | 1.81 | 4.99 | 14.22 | 44.31 | 124.30 | 345.27 | 990.29 |
|  | ng/mL | 0.55 | 2.00 | 5.35 | 15.38 | 44.90 | 116.57 | 368.31 | 1029.48 |
| 610092 | ng/mL | 0.36 | 1.69 | 5.26 | 16.59 | 48.00 | 126.01 | 379.26 | 1147.40 |
|  | ng/mL | 0.44 | 1.49 | 5.14 | 15.19 | 45.15 | 123.67 | 391.21 | 1184.79 |
| Mean value, n=6 | ng/mL | 0.51 | 1.73 | 5.08 | 15.19 | 44.89 | 126.17 | 371.88 | 1081.43 |
| SD | ng/mL | 0.10 | 0.17 | 0.22 | 0.79 | 1.71 | 6.57 | 17.38 | 73.43 |
| CV | % | 19 | 10 | 4 | 5 | 4 | 5 | 5 | 7 |
| Nominal value | ng/mL | 0.54 | 1.61 | 4.83 | 14.49 | 43.47 | 130.40 | 391.21 | 1173.62 |
| Accuracy | % | -5 | 7 | 5 | 5 | 3 | -3 | -5 | -8 |
| TE | % | 24 | 17 | 9 | 10 | 7 | 8 | 10 | 15 |

**Table 3** Intra batch accuracy and precision for TAP QC samples (n=6)

| Run 610090 | Unit | QC1 | QC2 | QC3 |
| --- | --- | --- | --- | --- |
| Measurement 1 | ng/mL | 26.98 | 304.58 | 472.26 |
| Measurement 2 | ng/mL | 25.20 | 292.41 | 489.80 |
| Measurement 3 | ng/mL | 25.37 | 309.46 | 481.38 |
| Measurement 4 | ng/mL | 26.01 | 281.57 | 479.37 |
| Measurement 5 | ng/mL | 25.80 | 310.66 | 467.56 |
| Measurement 6 | ng/mL | 26.29 | 290.68 | 495.66 |
| Mean value, n=6 | ng/mL | 25.94 | 298.23 | 481.01 |
| SD | ng/mL | 0.65 | 11.74 | 10.51 |
| CV | % | 2 | 4 | 2 |
| Nominal value | ng/mL | 25.66 | 316.32 | 468.34 |
| Accuracy | % | 1 | -6 | 3 |
| TE | % | 4 | 10 | 5 |

**Table 4** Inter batch accuracy and precision for TAP QC samples (n=6)

| Run | Unit | QC1 | QC2 | QC3 |
| --- | --- | --- | --- | --- |
| 610090 | ng/mL | 25.42 | 322.51 | 484.27 |
|  | ng/mL | 26.31 | 318.53 | 478.06 |
| 610091 | ng/mL | 24.95 | 304.46 | 435.77 |
|  | ng/mL | 25.46 | 298.13 | 426.97 |
| 610092 | ng/mL | 27.81 | 356.13 | 462.12 |
|  | ng/mL | 28.17 | 339.23 | 500.17 |
| Mean value, n=6 | ng/mL | 26.35 | 323.17 | 464.56 |
| SD | ng/mL | 1.35 | 21.65 | 28.59 |
| CV | % | 5 | 7 | 6 |
| Nominal value | ng/mL | 25.66 | 316.32 | 468.34 |
| Accuracy | % | 3 | 2 | -1 |
| TE | % | 8 | 9 | 7 |

### CPA1 accuracy and precision

**Table 5** Overview of accuracy and precision experiments for CPA1 (n=6)

| Sample set | Parameter | Acceptance criteria | Range within criteria | Min (%) | Max (%) |
| --- | --- | --- | --- | --- | --- |
| S1-S8 | Inter batch precision (%CV) | ≤20%, ≤ 25% at LLOQ | S1-S7 | 9 | 12 |
| S1-S8 | Inter batch accuracy (%accuracy) | ≤±20% | S1-S8 | -12 | 14 |
| S1-S8 | Inter batch total error (%TE) | ≤40% | S1-S7 | 11 | 26 |
| QC1-3 | Intra batch precision (%CV) | ≤20%, ≤ 25% at LLOQ | QC1-3 | 5 | 6 |
| QC1-3 | Intra batch accuracy (%accuracy) | ≤±20% | QC1-3 | -1 | 4 |
| QC1-3 | Intra batch total error (%TE) | ≤40% | QC1-3 | 7 | 9 |
| QC1-3 | Inter batch precision (%CV) | ≤20%, ≤ 25% at LLOQ | QC1-3 | 12 | 14 |
| QC1-3 | Inter batch accuracy (%accuracy) | ≤±20% | QC1-3 | -7 | 5 |
| QC1-3 | Inter batch total error (%TE) | ≤40% | QC1-3 | 12 | 20 |

**Table 6** Inter batch accuracy and precision, calibration samples CPA1 (n=6)

| Run | Unit | S8 | S7 | S6 | S5 | S4 | S3 | S2 | S1 |
| --- | --- | --- | --- | --- | --- | --- | --- | --- | --- |
| 610090 | ng/mL | 7.09 | 25.02 | 77.80 | 258.29 | 647.07 | 1880.10 | 5284.27 | 17147.39 |
|  | ng/mL | 5.86 | 32.15 | 77.97 | 219.08 | 682.27 | 1839.54 | 5728.94 | 17630.29 |
| 610091 | ng/mL | 8.93 | 25.63 | 86.56 | 254.26 | 769.91 | 2304.96 | 6113.59 | 17909.68 |
|  | ng/mL | 12.65 | 32.87 | 79.78 | 277.59 | 810.98 | 2177.88 | 6387.53 | 17380.06 |
| 610092 | ng/mL | 8.62 | 31.39 | 63.60 | 204.01 | 642.82 | 1801.66 | 5155.50 | 14841.60 |
|  | ng/mL | 6.03 | 30.76 | 75.16 | 211.78 | 604.30 | 1729.49 | 5149.00 | 14723.50 |
| Mean value, n=6 | ng/mL | 8.20 | 29.64 | 76.81 | 237.50 | 692.89 | 1955.60 | 5636.47 | 16605.42 |
| SD | ng/mL | 2.53 | 3.42 | 7.53 | 29.81 | 80.56 | 230.41 | 527.88 | 1435.13 |
| CV | % | 31 | 12 | 10 | 13 | 12 | 12 | 9 | 9 |
| Nominal value | ng/mL | 8.63 | 25.90 | 77.69 | 233.07 | 699.21 | 2097.64 | 6292.93 | 18878.80 |
| Accuracy | % | -5 | 14 | -1 | 2 | -1 | -7 | -10 | -12 |
| TE | % | 36 | 26 | 11 | 14 | 13 | 19 | 20 | 21 |

**Table 7** Intra batch accuracy and precision for CPA1 QC samples (n=6)

| Run 610090 | Unit | QC1 | QC2 | QC3 |
| --- | --- | --- | --- | --- |
| Measurement 1 | ng/mL | 405.21 | 2958.11 | 5605.11 |
| Measurement 2 | ng/mL | 378.39 | 3197.37 | 6002.96 |
| Measurement 3 | ng/mL | 373.42 | 3074.64 | 5868.25 |
| Measurement 4 | ng/mL | 347.49 | 2958.80 | 5321.70 |
| Measurement 5 | ng/mL | 367.55 | 3101.50 | 5259.29 |
| Measurement 6 | ng/mL | 364.39 | 2708.34 | 5902.14 |
| Mean value, n=6 | ng/mL | 372.74 | 2999.80 | 5659.91 |
| SD | ng/mL | 19.08 | 169.32 | 315.48 |
| CV | % | 5 | 6 | 6 |
| Nominal value | ng/mL | 365.17 | 2895.39 | 5737.89 |
| Accuracy | % | 2 | 4 | -1 |
| TE | % | 7 | 9 | 7 |

**Table 8** Inter batch accuracy and precision for CPA1 QC samples (n=6)

| Run | Unit | QC1 | QC2 | QC3 |
| --- | --- | --- | --- | --- |
| 610090 | ng/mL | 372.93 | 3049.73 | 4854.31 |
|  | ng/mL | 370.81 | 3182.55 | 5358.66 |
| 610091 | ng/mL | 395.82 | 3638.64 | 6207.11 |
|  | ng/mL | 424.34 | 3291.08 | 6332.50 |
| 610092 | ng/mL | 321.67 | 2586.90 | 4721.92 |
|  | ng/mL | 310.60 | 2519.01 | 4704.34 |
| Mean value, n=6 | ng/mL | 366.03 | 3044.65 | 5363.14 |
| SD | ng/mL | 43.35 | 428.59 | 742.44 |
| CV | % | 12 | 14 | 14 |
| Nominal value | ng/mL | 365.17 | 2895.39 | 5737.89 |
| Accuracy | % | 0 | 5 | -7 |
| TE | % | 12 | 19 | 20 |

### CPA2 accuracy and precision

**Table 9** Overview of accuracy and precision experiments for CPA2 (n=6)

| Sample set | Parameter | Acceptance criteria | Range within criteria | Min (%) | Max (%) |
| --- | --- | --- | --- | --- | --- |
| S1-S8 | Inter batch precision (%CV) | ≤20%, ≤ 25% at LLOQ | S1-S8 | 2 | 19 |
| S1-S8 | Inter batch accuracy (%accuracy) | ≤±20% | S1-S8 | -4 | 5 |
| S1-S8 | Inter batch total error (%TE) | ≤40% | S1-S7 | 5 | 21 |
| QC1-3 | Intra batch precision (%CV) | ≤20%, ≤ 25% at LLOQ | QC1-3 | 7 | 9 |
| QC1-3 | Intra batch accuracy (%accuracy) | ≤±20% | QC1-3 | -5 | 0 |
| QC1-3 | Intra batch total error (%TE) | ≤40% | QC1-3 | 9 | 13 |
| QC1-3 | Inter batch precision (%CV) | ≤20%, ≤ 25% at LLOQ | QC1-3 | 6 | 10 |
| QC1-3 | Inter batch accuracy (%accuracy) | ≤±20% | QC1-3 | -6 | 16 |
| QC1-3 | Inter batch total error (%TE) | ≤40% | QC1-3 | 13 | 26 |

**Table 10** Overview of accuracy and precision experiments for CPA2 (n=6)

| Run | Unit | S8 | S7 | S6 | S5 | S4 | S3 | S2 | S1 |
| --- | --- | --- | --- | --- | --- | --- | --- | --- | --- |
| 1 | ng/mL | 10.08 | 20.75 | 65.61 | 210.88 | 720.05 | 2183.47 | 6247.88 | 18317.03 |
|  | ng/mL | 11.37 | 23.06 | 68.32 | 187.23 | 681.02 | 2146.17 | 6334.32 | 17640.27 |
| 2 | ng/mL | 7.82 | 19.35 | 87.60 | 273.17 | 725.86 | 2260.36 | 6504.43 | 18424.14 |
|  | ng/mL | 12.62 | 27.67 | 80.57 | 268.94 | 822.46 | 2173.83 | 6455.34 | 18494.78 |
| 3 | ng/mL | 8.56 | 28.61 | 84.76 | 233.77 | 702.02 | 2223.50 | 6483.40 | 18174.85 |
|  | ng/mL | 8.59 | 29.47 | 72.05 | 281.56 | 714.69 | 2299.10 | 7086.76 | 17922.47 |
| Mean value, n=6 | ng/mL | 9.84 | 24.82 | 76.48 | 242.59 | 727.68 | 2214.40 | 6518.69 | 18162.26 |
| SD | ng/mL | 1.87 | 4.33 | 9.09 | 38.20 | 49.09 | 57.67 | 295.12 | 326.57 |
| CV | % | 19 | 17 | 12 | 16 | 7 | 3 | 5 | 2 |
| Nominal value | ng/mL | 8.58 | 25.74 | 77.22 | 231.66 | 694.99 | 2084.98 | 6254.93 | 18764.80 |
| Accuracy | % | 15 | -4 | -1 | 5 | 5 | 6 | 4 | -3 |
| TE | % | 34 | 21 | 13 | 20 | 11 | 9 | 9 | 5 |

**Table 11** Intra batch accuracy and precision for CPA2 QC samples (n=6)

|  | Unit | QC1 | QC2 | QC3 |
| --- | --- | --- | --- | --- |
| Measurement 1 | ng/mL | 52.99 | 598.00 | 1119.58 |
| Measurement 2 | ng/mL | 59.94 | 578.88 | 1068.43 |
| Measurement 3 | ng/mL | 53.12 | 550.66 | 1024.27 |
| Measurement 4 | ng/mL | 62.07 | 501.37 | 938.58 |
| Measurement 5 | ng/mL | 59.82 | 512.54 | 942.27 |
| Measurement 6 | ng/mL | 49.65 | 467.18 | 1054.80 |
| Mean value, n=6 | ng/mL | 56.26 | 534.77 | 1024.66 |
| SD | ng/mL | 4.98 | 49.75 | 72.16 |
| CV | % | 9 | 9 | 7 |
| Nominal value | ng/mL | 56.17 | 555.79 | 1074.74 |
| Accuracy | % | 0 | -4 | -5 |
| TE | % | 9 | 13 | 12 |

**Table 12** Inter batch accuracy and precision for CPA2 QC samples (n=6)

| Run | Unit | QC1 | QC2 | QC3 |
| --- | --- | --- | --- | --- |
| 1 | ng/mL | 66.80 | 593.78 | 969.51 |
|  | ng/mL | 52.62 | 566.12 | 943.18 |
| 2 | ng/mL | 66.44 | 640.05 | 1134.94 |
|  | ng/mL | 69.22 | 652.18 | 1020.12 |
| 3 | ng/mL | 71.41 | 591.37 | 950.71 |
|  | ng/mL | 63.28 | 581.86 | 1050.33 |
| Mean value, n=6 | ng/mL | 64.96 | 604.23 | 1011.46 |
| SD | ng/mL | 6.64 | 34.09 | 73.41 |
| CV | % | 10 | 6 | 7 |
| Nominal value | ng/mL | 56.17 | 555.79 | 1074.74 |
| Accuracy | % | 16 | 9 | -6 |
| TE | % | 26 | 14 | 13 |

**Table 13** Stability assessment for TAP QC samples

|  | Storage |  | Unit | Sample | | |
| --- | --- | --- | --- | --- | --- | --- |
|  | temperature |  |  | QC1 (unspiked) | QC2 (unspiked) | QC3 (unspiked) |
|  |  | Nominal value | ng/mL | 25.66 | 316.32 | 468.34 |
| Freeze/thaw stability | -80°C | 1x Freeze/thaw | ng/mL | 27.27 | 361.03 | 527.11 |
|  |  | Accuracy | % | 6 | 14 | 13 |
| Freeze/thaw stability | -80°C | 2x Freeze/thaw | ng/mL | 26.02 | 370.63 | 512.58 |
|  |  | Accuracy | % | 1 | 17 | 9 |
| Freeze/thaw stability | -80°C | 3x Freeze/thaw | ng/mL | 28.20 | 362.66 | 521.72 |
|  |  | Accuracy | % | 10 | 15 | 11 |
| Bench-top stability | RT | 2 h stability | ng/mL | 29.05 | 348.01 | 558.68 |
|  |  | Accuracy | % | 13 | 10 | 19 |
| Bench-top stability | RT | 24 h stability | ng/mL | 29.89 | 357.14 | 533.65 |
|  |  | Accuracy | % | 16 | 13 | 14 |
| Freeze/thaw stability digest | -20°C | 1x Freeze/thaw | ng/mL | 27.05 | 360.44 | 521.87 |
|  |  | Accuracy | % | 5 | 14 | 11 |
| Auto sampler stability | 5°C | 24 h in autosampler | ng/mL | 25.85 | 335.87 | 495.41 |
|  |  | Accuracy | % | 1 | 6 | 6 |
| Auto sampler stability | 5°C | 48 h in autosampler | ng/mL | 27.43 | 328.83 | 493.68 |
|  |  | Accuracy | % | 7 | 4 | 5 |
| Auto sampler stability | 5°C | 72 h in autosampler | ng/mL | 24.02 | 318.87 | 443.51 |
|  |  | Accuracy | % | -6 | 1 | -5 |

**Table 14** Stability assessment for CPA1 QC samples

|  | Storage |  | Unit | Sample | | |
| --- | --- | --- | --- | --- | --- | --- |
|  | temperature |  |  | QC1 (unspiked) | QC2 (unspiked) | QC3 (unspiked) |
|  |  | Nominal value | ng/mL | 365.17 | 2895.39 | 5737.89 |
| Freeze/thaw stability | -80°C | 1x Freeze/thaw | ng/mL | 331.06 | 2696.12 | 4365.77 |
|  |  | Accuracy | % | -9 | -7 | -24 |
| Freeze/thaw stability | -80°C | 2x Freeze/thaw | ng/mL | 289.85 | 2570.29 | 4481.88 |
|  |  | Accuracy | % | -21 | -11 | -22 |
| Freeze/thaw stability | -80°C | 3x Freeze/thaw | ng/mL | 303.45 | 2484.87 | 4489.41 |
|  |  | Accuracy | % | -17 | -14 | -22 |
| Bench-top stability | RT | 2 h stability | ng/mL | 322.90 | 2469.14 | 4536.22 |
|  |  | Accuracy | % | -12 | -15 | -21 |
| Bench-top stability | RT | 24 h stability | ng/mL | 314.61 | 2606.20 | 4566.36 |
|  |  | Accuracy | % | -14 | -10 | -20 |
| Freeze/thaw stability digest | -20°C | 1x Freeze/thaw | ng/mL | 417.44 | 3633.21 | 6383.48 |
|  |  | Accuracy | % | 14 | 25 | 11 |
| Auto sampler stability | 5°C | 24 h in autosampler | ng/mL | 374.74 | 3124.70 | 5351.58 |
|  |  | Accuracy | % | 3 | 8 | -7 |
| Auto sampler stability | 5°C | 48 h in autosampler | ng/mL | 309.81 | 2522.54 | 4739.83 |
|  |  | Accuracy | % | -15 | -13 | -17 |
| Auto sampler stability | 5°C | 72 h in autosampler | ng/mL | 413.28 | 3536.46 | 6335.91 |
|  |  | Accuracy | % | 13 | 22 | 10 |

**Table 15** Stability assessment for CPA2 QC samples

|  | Storage |  | Unit | Sample | | |
| --- | --- | --- | --- | --- | --- | --- |
|  | temperature |  |  | QC1 (unspiked) | QC2 (unspiked) | QC3 (unspiked) |
|  |  | Nominal value | ng/mL | 56.17 | 555.79 | 1074.74 |
| Freeze/thaw stability | -80°C | 1x Freeze/thaw | ng/mL | 57.60 | 535.04 | 962.79 |
|  |  | Accuracy | % | 3 | -4 | -10 |
| Freeze/thaw stability | -80°C | 2x Freeze/thaw | ng/mL | 62.93 | 519.25 | 909.11 |
|  |  | Accuracy | % | 12 | -7 | -15 |
| Freeze/thaw stability | -80°C | 3x Freeze/thaw | ng/mL | 66.76 | 533.45 | 962.38 |
|  |  | Accuracy | % | 19 | -4 | -10 |
| Bench-top stability | RT | 2 h stability | ng/mL | 56.44 | 570.82 | 965.69 |
|  |  | Accuracy | % | 0 | 3 | -10 |
| Bench-top stability | RT | 24 h stability | ng/mL | 55.91 | 547.53 | 959.56 |
|  |  | Accuracy | % | 0 | -1 | -11 |
| Freeze/thaw stability digest | -20°C | 1x Freeze/thaw | ng/mL | 68.17 | 684.18 | 1171.87 |
|  |  | Accuracy | % | 21 | 23 | 9 |
| Auto sampler stability | 5°C | 24 h in autosampler | ng/mL | 58.23 | 559.32 | 995.15 |
|  |  | Accuracy | % | 4 | 1 | -7 |
| Auto sampler stability | 5°C | 48 h in autosampler | ng/mL | 63.47 | 558.23 | 996.77 |
|  |  | Accuracy | % | 13 | 0 | -7 |
| Auto sampler stability | 5°C | 72 h in autosampler | ng/mL | 53.04 | 630.70 | 1062.80 |
|  |  | Accuracy | % | -6 | 13 | -1 |
